# Supplementary material for: Holocene climate change in southern Oman deciphered by speleothem records and climate model simulations
Source: Nat Commun. 2023 Aug 5;14:4718. doi: 10.1038/s41467-023-40454-z (PMC10404270; doi:10.1038/s41467-023-40454-z)
Supplement: Supplementary file 1 — Supplementary Information [file 41467_2023_40454_MOESM1_ESM.pdf]

Supplementary Information for

Holocene climate change in southern Oman deciphered by speleothem records and climate model simulations

Ye Tian[1], Dominik Fleitmann[2], Qiong Zhang[3], Lijuan Sha[1], Jasper. A. Wassenburg[4,5], Josefine Axelsson[3], Haiwei Zhang[1], Xianglei Li[6], Jun Hu[7], Hanying Li[1], Liang Zhao[8], Yanjun Cai[1], Youfeng Ning[1], Hai Cheng[1, 6]*

This PDF file includes:

Supplementary Discussion
Supplementary Table 1
Supplementary Figures 1 to 9
Supplementary References

Supplementary Discussion

Age-depth model comparison

There are several dating ages from Fleitmann, et al. [1] which appear to be a few hundred-year offset from this study's ^{230}Th dating results (Supplementary Fig. 1). Fleitmann et al. (2003) obtained 18 dating results mainly by TIMS, which need a large amount of sample for measuring. The diameter of the sampling holes could be 8 to 10mm, covering more than one layer of the stalagmite (Supplementary Fig. 3), this leads to very large dating errors. In our study, only ~50mg powder was drilled from each dating point and was taken only from one growth laminae, less than 2mm.

On the other hand, the dating method has greatly developed during the past two decades, and especially, Cheng et al.[2] have improved the ^{230}Th dating by MC-ICP-MS. On the basis of the improved techniques, both dating precision and dating resolution of Q5 are largely improved in this study. Nevertheless, the Q5 $\delta^{18}O_c$ and $\delta^{13}C$ records have no significant difference between the 2003 and 2023 age models, particularly in terms of their Holocene patterns.

Local hydroclimate changes

The Qunf $\delta^{18}O_c$ record generally reflects the large-scale monsoon circulation changes in addition to precipitation amount, but does not necessarily indicate changes in effective wetness (precipitation minus evaporation, P-E) at the cave site. Instead, signals of local P-E may be reconstructed from other proxies, such as $(^{234}U/^{238}U)_0$, $\delta^{13}C$, and trace element records[3,4]. In addition, data-model comparisons are also very useful to understand local hydroclimate changes. In the following discussions, we present some information related to the local hydrological changes, which may provide a primary basis for further work.

A number of studies have shown that the initial activity ratio of ^{234}U to ^{238}U of speleothems, $(^{234}U/^{238}U)_0$, may indicate the hydroclimate condition[4]. Three main factors that affect speleothem $(^{234}U/^{238}U)_0$ values are (1) preferential leaching of ^{234}U from the sites in crystal lattice damaged by the alpha decay of ^{238}U; (2) alpha recoil of ^{234}U into the water phase; and (3) the lower mass of ^{234}U and its higher mobility than ^{238}U. Basically, $(^{234}U/^{238}U)_0$ varies with the above factors that in turn change with the rate of water flow and water-rock interaction times[5-10]. The Q5 $(^{234}U/^{238}U)_0$ ranges between 0.90 and 0.95. The lower values occur during ~10-5.5-ka BP and increase

afterwards. The lower ($^{234}U/^{238}U)_0$ between ~10-5.5 ka BP may be explained by less preferential leaching of ^{234}U due to shorter water residence times and/or higher infiltration rates. In other words, the water flow rates might be higher, and thus ($^{234}U/^{238}U)_0$ was less close to the secular equilibrium value (($^{234}U/^{238}U)_0 = 1$). In contrast, the ($^{234}U/^{238}U)_0$ record suggests a low water flow rates in the epikarst after 5.5 ka BP, which may result in the ($^{234}U/^{238}U)_0$ values closer to the equilibrium value[4,11-13].

In general, speleothem $\delta^{13}C$ is largely controlled by local hydrological/biological processes at the cave site, such as changes in local moisture availability (or P-E), carbon derived from the decomposition of organic matter, CO_2 from vegetation, effective infiltration, dissolved inorganic carbon (DIC) in soil water, and prior calcite precipitation (PCP) in the epikarst. Wet conditions are often associated with increased vegetation coverage, enhanced soil microbial productivity, high drip rate (rapid infiltration), reduced degassing of CO_2 from dripwater, and less PCP, which all tends to lead to more negative $\delta^{13}C$ values, and vice versa[14-21]. In this interpretation framework, the Q5 $\delta^{13}C$ record appears to suggest a relatively dry (lower P-E) to relatively wet (higher P-E) change across the Holocene as inferred from the $\delta^{13}C$ decrease trend.

PCP has often been considered a common process in the vadose zone of many cave systems, affecting Mg/Ca, Sr/Ca and Ba/Ca ratios in cave drip waters. PCP increases with a reduction in effective infiltration[18,22-28]. Under drier conditions, reduced water recharge of the karst aquifer may lead to more intensive CO_2 degassing in the epikarst and thus PCP, resulting in progressive enrichment of Mg, Sr, and Ba relative to Ca in the seepage water, and subsequently in speleothems[22,27,29,30]. If PCP is the dominant process affecting speleothem trace element to Ca ratios, Mg/Ca, Sr/Ca and Ba/Ca ratios should correlate positively and the slope of the trend line in a natural log-log plot for Sr/Ca versus Mg/Ca should be between 0.709 and 1.45[29,31,32]. The correlation coefficients between Q5 Mg/Ca – Ba/Ca, Mg/Ca – Sr/Ca, and Ba/Ca – Sr/Ca Q5 are 0.871, 0.930, and 0.928, respectively. The slopes of the regression lines in the ln (Mg/Ca) *versus* ln (Sr/Ca) plot is 1.33 (Supplementary Fig. 6a), indicating that PCP can explain the speleothem Mg/Ca and Sr/Ca ratios. The slope of the trendline is directly controlled by the partitioning coefficients of the trace elements during carbonate precipitation. The slope of 1.33 for ln (Mg/Ca) *versus* ln (Sr/Ca) is a slope that can occur in environments with higher drip water Mg/Ca ratios as is the case at the Qunf Cave site. The higher Mg/Ca ratios in the calcite induce an increase in the Sr partitioning coefficient[29]. In low Mg/Ca ratio settings Sr and Ba calcite partitioning coefficients

are similar, i.e. around 0.11, resulting in a slope of about 1.0. In our case, the slope of the trendline in a plot of ln (Sr/Ca) *versus* ln (Ba/Ca) is around 0.7 (Supplementary Fig. 6b). However, the increase in Sr partitioning at the Qunf Cave will affect the slope. In addition, the effect of Mg/Ca ratios on the Ba partitioning coefficient in cave environments remains to be studied. The slope of the trendline in a plot of ln (Sr/Ca) *versus* ln (Ba/Ca) may thus be considerably different from 1 in high Mg/Ca settings. The slope of 1.33 for ln (Mg/Ca) *versus* ln (Sr/Ca) thus indicates potential a role for PCP in controlling Q5 trace element to Ca ratios. Collectively, the PCP (lower trace element ratios) after 6 ka BP may suggest a slightly higher P-E (or moisture availability) in the region, probably due to the reduced summer temperature and thus less evaporation under weakened summer insolation conditions. The observation that the trace element ratios are very low without a clear trend may indicate that the P-E condition does not change significantly as revealed in the simulation result (Supplementary Fig. 7). Or there might be a threshold of the P-E condition for the trace element ratios, above which the very low-level trace element ratios may not be sensitive to P-E changes. On the other hand, Qunf Cave is ~15 km away from the coastal line of the western Arabian Sea, and it is possible that sea spray is another source of trace elements[10,32,33]. In principle, sea spray could potentially increase salts in the local groundwater at the Qunf Cave site. In order to assess whether the sea spray was indeed a major source of the Q5 trace elements, further study is critically needed, including the comparison between Q5 and seawater trace element ratios. At Bunker Cave in Germany, a similar scenario involving an unidentified source of trace elements in the cave system was discussed by Folmeister et al.[34]. They suggested that windblown dust on the soil was progressively weathering and decalcifying, resulting in persistent elution of the aeolian deposits and decreasing Mg/Ca, Sr/Ca, and Ba/Ca ratios in the drip water and stalagmite.

Our simulation results show that both the North African summer monsoon and Indian summer monsoon were much stronger at 8K than at PI. The summer precipitation amount at Qunf Cave was marginally higher at 8K, while the summer P-E appears to be slightly lower over a large part of the Arabian Peninsula, suggesting a strong evaporation condition (Fig. 3, Supplementary Fig. 7).

However, given the variety of factors and multiplicity of proxy interpretations, further studies remain a prerequisite to understanding the Holocene hydroclimate history in the region.

Triple oxygen isotope analysis

$\Delta'^{17}O$ is calculated following equation[35,36]:

$$\Delta'^{17}O = \left(\delta'^{17}O_c - 0.528 \times \delta'^{18}O_c\right) \times 1000 \quad (1)$$

where $\delta'^{17}O_c = 1000 \times \ln\left(1 + \delta^{17}O_c / 1000\right)$ and $\delta'^{18}O = 1000 \times \ln\left(1 + \delta^{18}O_c / 1000\right)$. 0.528 is the slope of the triple oxygen isotope Global Meteoric Water Line.

To calculate relative humidity, isotopic compositions of carbonate parent water need to be reconstructed, the isotopic fractionations between paired cave dripwater and the carbonate precipitation from the water are needed, which is defined as[37]:

$$^{*}\alpha_{carbonate/water} = \left(\delta^{*}O_c + 1000\right) / \left(\delta^{*}O_{water} + 1000\right) \quad (2)$$

where $^{*}\alpha_{carbonate/water}$ stands for $^{18}\alpha_{carbonate/water}$ and $^{17}\alpha_{carbonate/water}$, and the exponent $\theta_{carbonate/water}$ is defined as[37]:

$$\theta_{carbonate/water} = \ln\left(^{17}\alpha_{carbonate/water}\right) / \ln\left(^{18}\alpha_{carbonate/water}\right) \quad (3)$$

where the value of $\theta_{carbonate/water}$ used in the study is 0.525. $^{18}\alpha_{carbonate/water}$ is defined by temperature following the equation[38]:

$$1000 \ln^{18}\alpha_{carbonate/water} = 18.03\left(10^3 T^{-1}\right) - 32.42 \quad (4)$$

where the temperature (T) is calculated from summer SST from ODP site 723A[39]. The relative humidity (h_n) is given by the following equations[40]:

$$\Delta'^{17}O(v) = -\ln\left(^{18}\alpha_{eq}^{0.529}\left(^{18}\alpha_{diff}^{0.518}\left(1 - h_n\right) + h_n\right)\right) + 0.528 \times \ln\left(^{18}\alpha_{eq}\left(^{18}\alpha_{diff}\left(1 - h_n\right) + h_n\right)\right) \quad (5)$$

where $\Delta'^{17}O(v)$ is the $\Delta'^{17}O$ in ocean surface water vapor. $^{18}\alpha_{eq}$ and $^{18}\alpha_{diff}$ are the $^{18}O/^{16}O$ isotope fractionation factor for liquid water-water equilibrium and diffusion, respectively. The value of $^{18}\alpha_{eq}$ used in the study is 1.0094, and the $^{18}\alpha_{diff}$ value is 1.0096 ±0.0018[37].

Two additional factors need to be corrected for $\Delta'^{17}O(v)$: 1. to correct the difference between seawater and VSMOW, 5 per meg ($\Delta'^{17}O_{(ocean/VSMOW)}$) need to be added to the estimated $\Delta'^{17}O$ values of speleothem parent water[41]; 2. vapor $\Delta'^{17}O$ value needs to be corrected for the closure

assumption bias ($\Delta_{closure}$ = 3 per meg)[42]. Thus the final corrected value, $\Delta'^{17}O(f)$, is given by the following equation[42]:

$$\Delta'^{17}O\ (f) = \Delta'^{17}O_{(parent\ water)} + \Delta_{closure} + \Delta'^{17}O_{(ocean/VSMOW)})\quad (6)$$

Individual errors are standard errors of the corresponding measurements propagated from $\delta^{17}O_c$ and $\delta^{18}O_c$ of speleothem carbonates[37,43]. The triple oxygen isotope compositions of parent water are calculated by the Monte Carlo model (MC=1000) in combination with statistical estimates of uncertainty.

Supplementary Table 1. Forcing and boundary conditions in pre-industrial (PI) and 8 ka BP (8K) experiments

	PI	8K
Eccentricity	0.016764	0.018386
Obliquity (degrees)	23.549	24.013
Perihelion – 180(degrees)	100.33	19.83
CO_2 (ppm)	284.3	283.3
CH_4 (ppb)	808.2	607.9
N_2O (ppb)	273.0	275.8
Topography and land-sea mask	Modern	Modern
Ice sheets	Modern	Modern
Vegetation	CMIP DECK PI	CMIP DECK PI

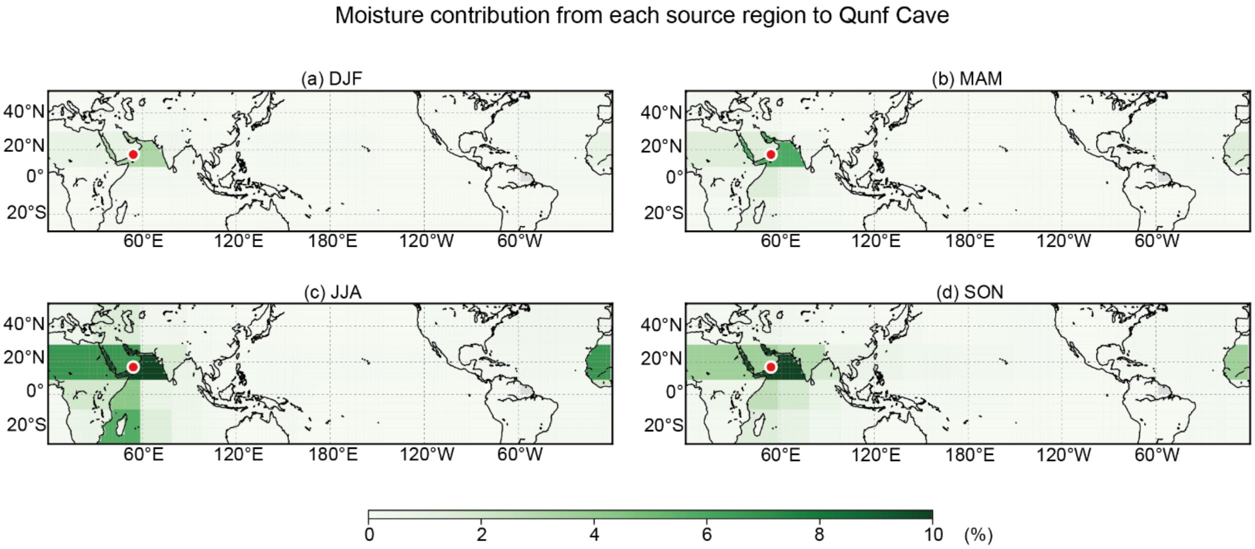

Supplementary Fig. 1. Seasonal moisture contribution from each source to Qunf Cave in the iCESM simulation during 1954-2012[44]

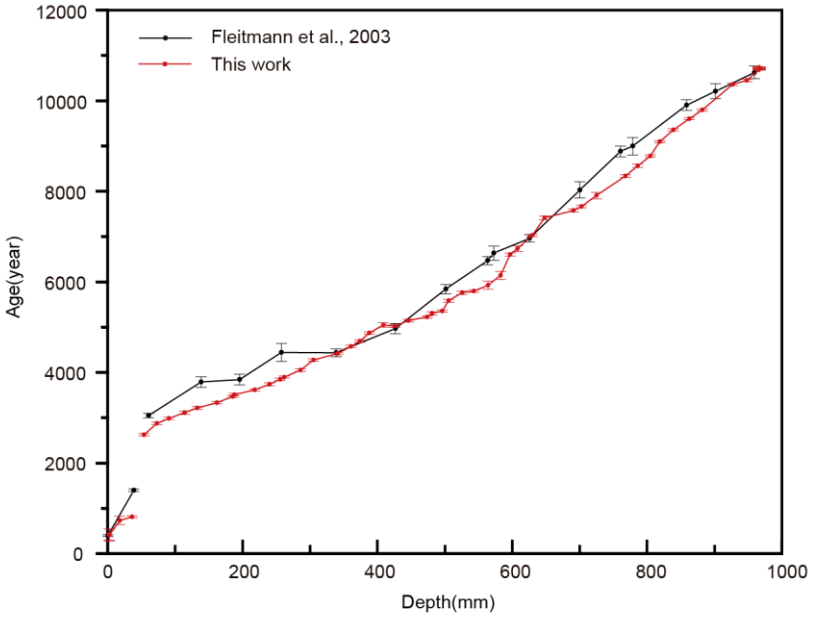

Supplementary Fig. 2. Plot of age versus depth for stalagmite Q5 in this study (red) compared with the age model in Fleitmann et al.[1] (black). All errors are 2σ.

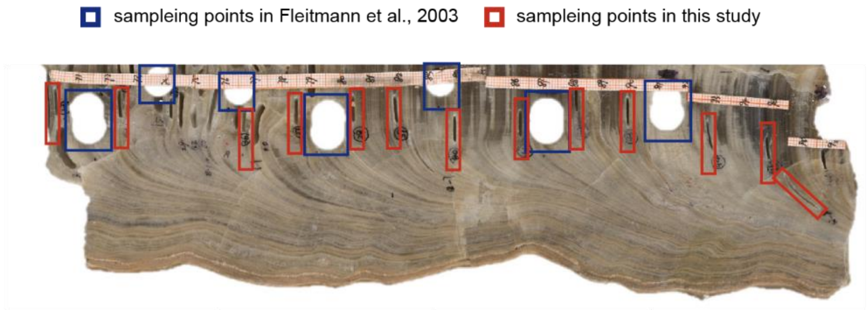

Supplementary Fig. 3. Comparison of stalagmite Q5 sampling points between Fleitmann et al. (2003) and this study

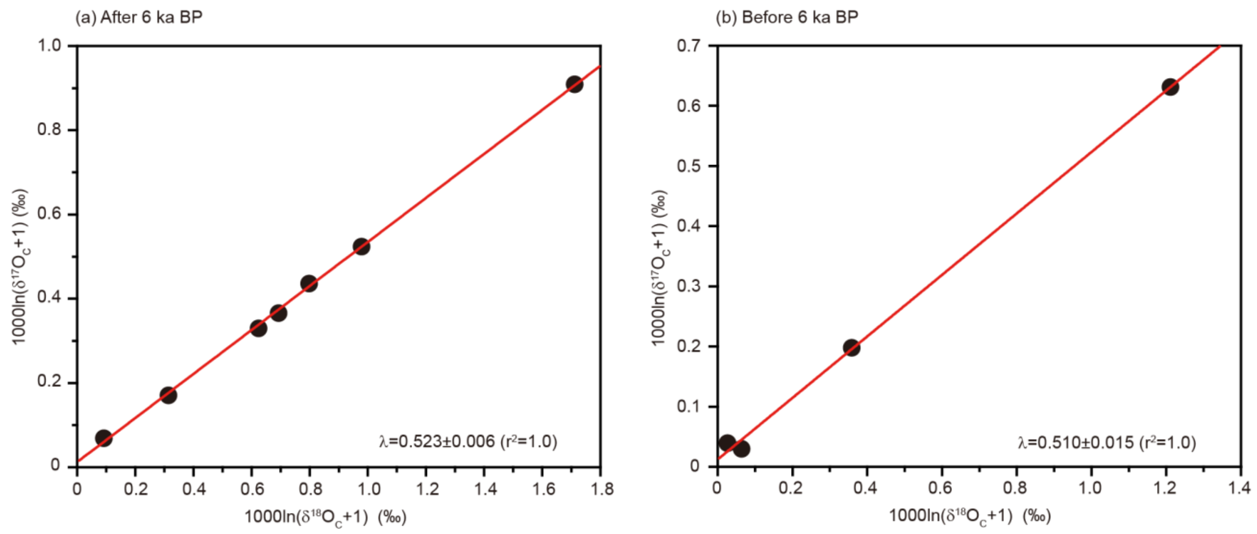

Supplementary Fig. 4. **Variation of Q5 $\delta^{18}O_c$-$\delta^{17}O_c$ fractionation during the Holocene.** The slope of the $1000\ln(\delta^{18}O_c+1) - 1000\ln(\delta^{17}O_c+1)$ line is (**a**) 0.523 ± 0.015 (r^2=1.0) from 6 ka BP to the present and (**b**) 0.510 ± 0.006 (r^2=1.0) between 10.8 – 6 ka BP. Indicating a change of moisture at 6 ka BP.

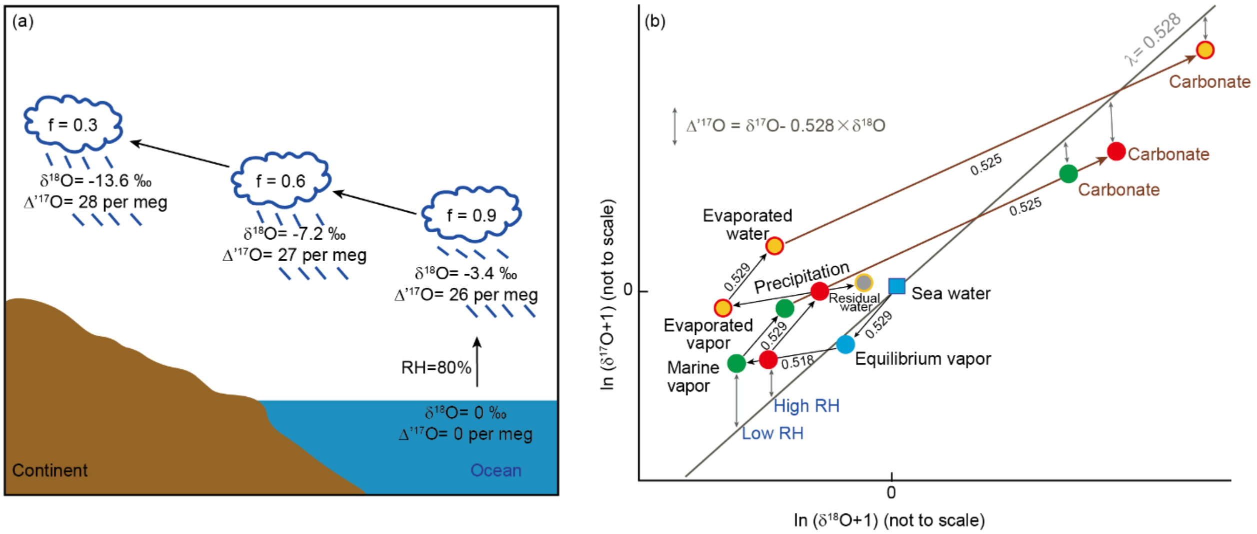

Supplementary Fig. 5. **Variation of liquid $\delta^{18}O$ and $\Delta'^{17}O$ during Rayleigh distillation in (a) a schematic and (b) $\delta^{18}O$–$\delta^{17}O$ isotope space.** (a) Water starts in the ocean, evaporates, and condenses, leaving airmasses with 90%, 60%, and 30% of the initial airmass remaining. f is the percentage of the initial airmass that remains after rainout. The isotopic composition of precipitation was calculated at 25℃ and is assumed to be in isotopic equilibrium with the vapor. (b) Generalized pathway of $\delta^{18}O$ and $\delta^{17}O$ fractionation in the water cycle. $\Delta'^{17}O$ is defined as the deviation from the reference line. Water at different points in the hydrologic cycle is differentiated with symbols associated with evaporation and condensation. Beginning from the ocean, water evaporates into a saturated equilibrium layer, diffuses through the unsaturated atmosphere, condenses, and evaporates. The symbols do not indicate end points, instead, show points on a trajectory (marked with arrows) along which isotopic compositions can continue.

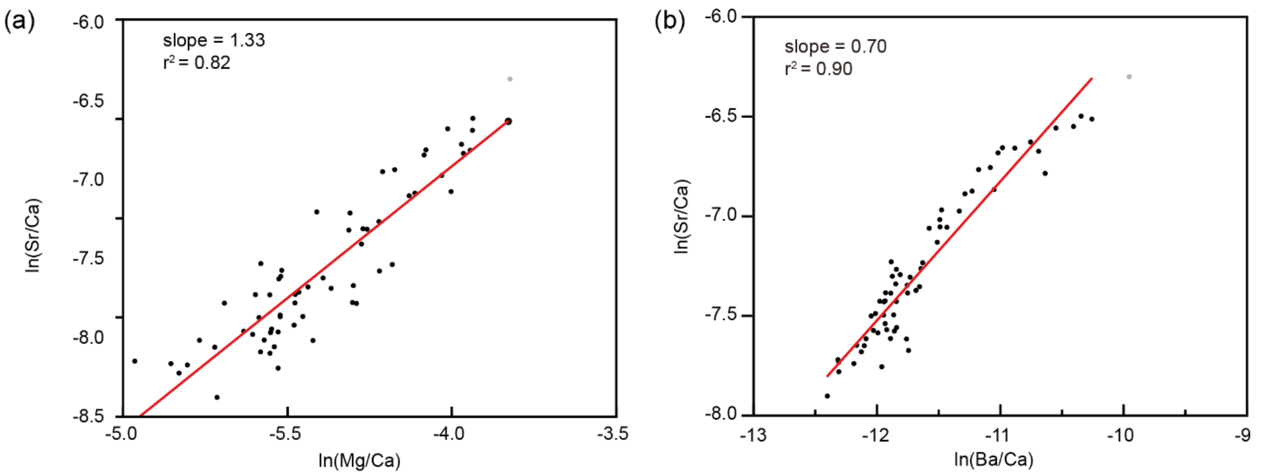

Supplementary Fig. 6. (**a**) ln (Sr/Ca) vs ln (Mg/Ca) and (**b**) ln (Sr/Ca) vs ln (Ba/Ca) for speleothem Q5. After removing the abnormal data (grey dots), the slopes of regression lines in the ln (Mg/Ca) vs ln (Sr/Ca) and ln (Ba/Ca) vs ln (Sr/Ca) plots are 1.33 and 0.70, within or close to the theoretical range of PCP process which is between 0.709 and 1.45.

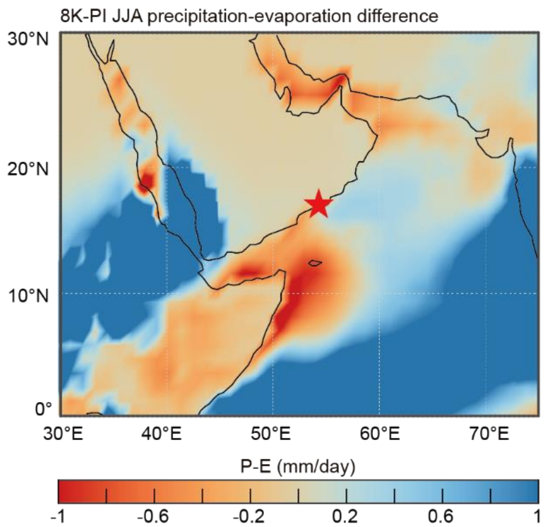

Supplementary Fig. 7. EC-Earth simulated June-July-August (JJA) precipitation minus evaporation (P-E) difference between 8 ka BP (8K) and pre-industrial period (PI). The results show that P-E was unchanged or slightly lower in 8K compared with PI over most of the Arabia Peninsula, including the cave site (red asterisk).

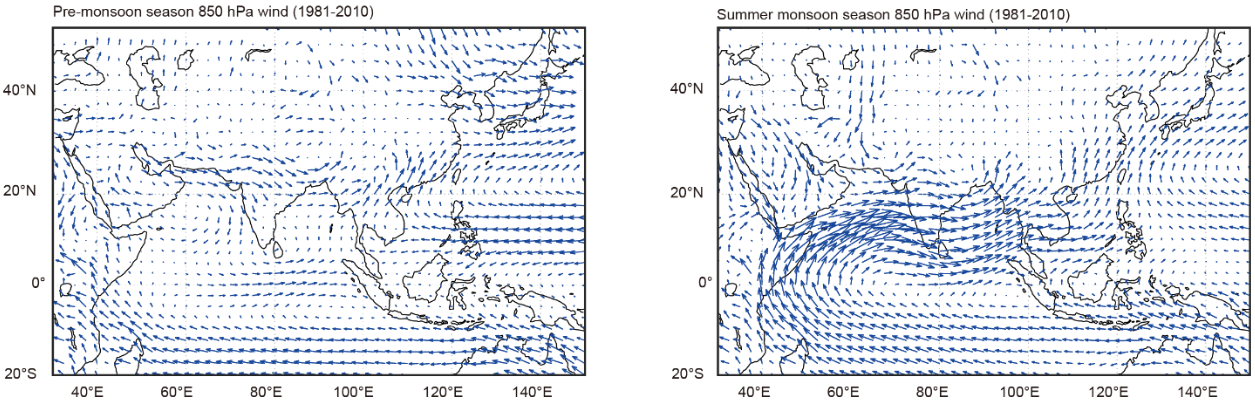

Supplementary Figure 8. **850 hPa wind** of (**a**) pre-monsoon season (March to May, MAM), and (**b**) monsoon season (June to July) during 1981-2010 from ERA5[45].

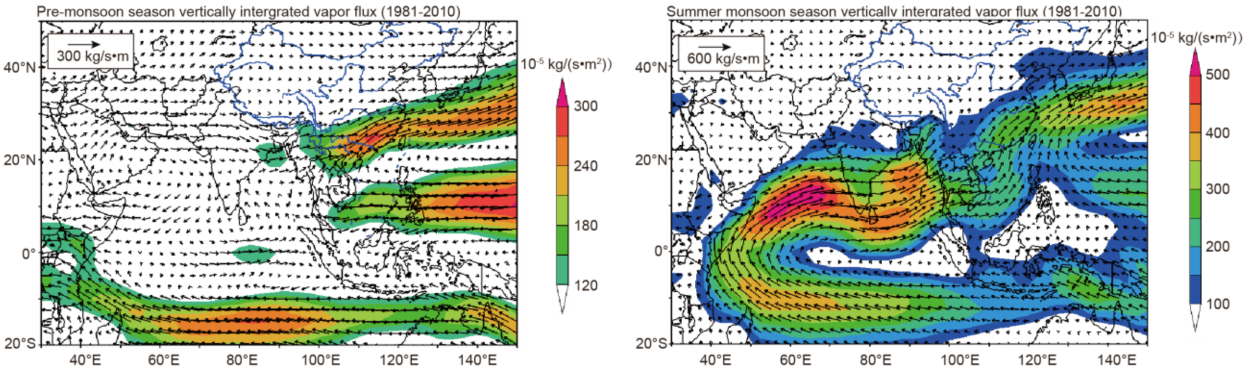

Supplementary Figure 9. Vertically integrated vapor flux of (**a**) pre-monsoon season (March 15 to May 15), and (**b**) monsoon season (June 1 to July 15) during 1981-2010 from Nation Climate Centre of China.

Supplementary References

1. Fleitmann, D. et al. Holocene Forcing of the Indian Monsoon Recorded in a Stalagmite from Southern Oman. *Science* **300**, 1737-1739 (2003).2 Zhang, H. et al. A data-model comparison pinpoints Holocene spatiotemporal pattern of East Asian summer monsoon. *Quat. Sci. Rev.* **261**, 106911 (2021).
2. Cheng, H. *et al.* Improvements in ^{230}Th dating, ^{230}Th and ^{234}U half-life values, and U–Th isotopic measurements by multi-collector inductively coupled plasma mass spectrometry. *Earth Planet. Sci. Lett.* **371-372**, 82-91 (2013).
3. Zhang, H. et al. A data-model comparison pinpoints Holocene spatiotemporal pattern of East Asian summer monsoon. *Quat. Sci. Rev.* 261, doi:10.1016/j.quascirev.2021.106911 (2021).
4. Hellstrom, J. C. & McCulloch, M. T. Multi-proxy constraints on the climatic significance of trace element records from a New Zealand speleothem. *Earth Planet. Sci. Lett.* **179**, 287-297 (2000).
5. Hercman, H., Błaszczyk, M., Mulczyk, A. & Bella, P. Uranium isotopic ratios and their implication for uranium–uranium dating and groundwater circulation studies: A case study from speleothems of the Demänová caves, Nízke Tatry Mts., Slovakia. *Geologica Carpathica* **71**, 61-72 (2020).
6. Osmond, J.K. U-series nuclides as tracers in groundwater hydrology. In: Environmental Tracers in Subsurface Hydrology. Kluwer Academic Publishers, Boston, (2000).
7. Cherdyntsev, V. V. Uranium-234. (John Wiley and Sons, Inc., New York, 1971).
8. Rosholt, J.N. Mobilization and weathering, in: Uranium Series Disequilibrium: Applications to Environmental Problems, Clarendon Press, Oxford, (1982).
9. Henderson, G. M., Slowey, N. C. & Haddad, G. A. Fluid flow through carbonate platforms: Constraints from ^{234}U/^{238}U and Cl^- in Bahamas pore-waters. *Earth Planet. Sci. Lett.* **169**, 99-111 (1999).
10. Ayalon, A., Bar-Matthews, M. & Kaufman, A. Petrography, strontium, barium and uranium concentrations, and strontium and uranium isotope ratios in speleothems as palaeoclimatic proxies: Soreq Cave, Israel. *The Holocene* **9**, 715-722 (1999).
11. Denniston, R. F. et al. Speleothem evidence for Holocene fluctuations of the prairie-forest ecotone, north-central USA. *Holocene* **9**, 671-676 (1999).
12. Kaufman, A. et al. U-Th isotope systematics from the Soreq cave, Israel and climatic correlations. *Earth Planet. Sci. Lett.* **156**, 141-155 (1998).
13. Zhou, J. et al. Geochemistry of speleothem records from southern Illinois: Development of (^{234}U)/(^{238}U) as a proxy for paleoprecipitation. *Chemical Geology* **221**, 1-20 (2005).
14. Bar-Matthews, M., Ayalon, A., Matthews, A., Sass, E. & Halicz, L. Carbon and oxygen isotope study of the active water-carbonate system in a karstic Mediterranean cave: Implications for paleoclimate research in semiarid regions. *Geochim. Cosmochim. Acta* **60**, 235-241 (1996).
15. Baker, A., Ito, E., Smart, P. L. & Mcewan, R. F. Elevated and variable values of 13C in speleothems in a British cave system. *Chem. Geol.* **136**, 263-270 (1997).
16. Genty, D. et al. Precise dating of Dansgaard–Oeschger climate oscillations in western Europe from stalagmite data. *Nature* **421**, 833-837 (2003).
17. Genty, D. et al. Timing and dynamics of the last deglaciation from European and North African delta δ^{13}C stalagmite profiles—comparison with Chinese and South Hemisphere stalagmites. *Quat. Sci. Rev.* **25**, 2118-2142 (2006).
18. Fairchild, I. J. & Treble, P. C. Trace elements in speleothems as recorders of environmental change. *Quat. Sci. Rev.* **28**, 449-468 (2009).
19. Fohlmeister, J. et al. Main controls on the stable carbon isotope composition of speleothems. *Geochim. Cosmochim. Acta* **279**, 67-87 (2020).
20. Li, T. Y. et al. Transportation characteristics of delta C-13 in the plants-soil-bedrock-cave system in Chongqing karst area. *Sci. China-Earth Sci.* **55**, 685-694 (2012).
21. Fleitmann, D. et al. Droughts and societal change: The environmental context for the emergence of Islam in late Antique Arabia. *Science* **376**, 1317-1321 (2022).
22. Fairchild, I. J. et al. Controls on trace element (Sr–Mg) compositions of carbonate cave waters: implications for speleothem climatic records. *Chem. Geol.* **166**, 255-269 (2000).
23. Cruz, F. W. et al. Evidence of rainfall variations in Southern Brazil from trace element ratios (Mg/Ca and Sr/Ca) in a Late Pleistocene stalagmite. *Geochim. Cosmochim. Acta* **71**, 2250-2263 (2007).
24. Griffiths, M. L. et al. Younger Dryas–Holocene temperature and rainfall history of southern Indonesia from δ^{18}O in speleothem calcite and fluid inclusions. *Earth Planet. Sci. Lett.* **295**, 30-36 (2010).

25. Johnson, K., Hu, C., Belshaw, N. & Henderson, G. Seasonal trace-element and stable-isotope variations in a Chinese speleothem: The potential for high-resolution paleomonsoon reconstruction. *Earth Planet. Sci. Lett.* **244**, 394-407 (2006).
26. Verheyden, S., Keppens, E., Fairchild, I. J., McDermott, F. & Weis, D. Mg, Sr and Sr isotope geochemistry of a Belgian Holocene speleothem: implications for paleoclimate reconstructions. *Chem. Geol.* **169**, 131-144 (2000).
27. Tremaine, D. M. & Froelich, P. N. Speleothem trace element signatures: A hydrologic geochemical study of modern cave dripwaters and farmed calcite. *Geochim. Cosmochim. Acta* **121**, 522-545 (2013).
28. Tadros, C. V. et al. ENSO-cave drip water hydrochemical relationship: a 7-year dataset from south-eastern Australia. *Hydrol. Earth Syst. Sci.* **20**, 4625-4640 (2016).
29. Wassenburg, J. A. et al. Calcite Mg and Sr partition coefficients in cave environments: Implications for interpreting prior calcite precipitation in speleothems. *Geochim. Cosmochim. Acta* **269**, 581-596 (2020).
30. Stoll, H. M., Muller, W. & Prieto, M. I-STAL, a model for interpretation of Mg/Ca, Sr/Ca and Ba/Ca variations in speleothems and its forward and inverse application on seasonal to millennial scales. *Geochem. Geophys. Geosyst.* **13** (2012).
31. Sinclair, D. J. Two mathematical models of Mg and Sr partitioning into solution during incongruent calcite dissolution: implications for dripwater and speleothem studies. *Chem. Geol.* **283**, 119-133 (2011).
32. Sinclair, D. J. et al. Magnesium and strontium systematics in tropical speleothems from the Western Pacific. *Chem. Geol.* **294-295**, 1-17 (2012).
33. Goede, A., McCulloch, M., McDermott, F. & Hawkesworth, C. Aeolian contribution to strontium and strontium isotope variations in a Tasmanian speleothem. *Chem. Geol.* **149**, 37-50 (1998).
34. Fohlmeister, J. et al. Bunker Cave stalagmites: an archive for central European Holocene climate variability. *Clim. Past* **8**, 1751-1764 (2012).
35. Passey, B. H. *et al.* Triple oxygen isotopes in biogenic and sedimentary carbonates. *Geochim. Cosmochim. Acta* **141**, 1-25 (2014).
36. Passey, B. H. & Ji, H. Y. Triple oxygen isotope signatures of evaporation in lake waters and carbonates: A case study from the western United States. *Earth Planet. Sci. Lett.* **518**, 1-12 (2019).
37. Sha, L. J. *et al.* A novel application of triple oxygen isotope ratios of speleothems. *Geochim. Cosmochim. Acta* **270**, 360-378 (2020).
38. Kim, S. T. & O'Neil, J. R. Equilibrium and nonequilibrium oxygen isotope effects in synthetic carbonates. *Geochim. Cosmochim. Acta* **61**, 3461-3475 (1997).
39. Naidu, P. D. & Malmgren, B. A. Seasonal sea surface temperature contrast between the Holocene and last glacial period in the western Arabian Sea (Ocean Drilling Project Site 723A): Modulated by monsoon upwelling. *Paleoceanography* **20** (2005).
40. Barkan, E. & Luz, B. Diffusivity fractionations of $H_2{}^{16}O/H_2{}^{17}O$ and $H_2{}^{16}O/H_2{}^{18}O$ in air and their implications for isotope hydrology. *Rapid Commun Mass Spectrom* **21**, 2999-3005 (2007).
41. Landais, A. *et al.* Combined measurements of 17Oexcess and d-excess in African monsoon precipitation: Implications for evaluating convective parameterizations. *Earth Planet. Sci. Lett.* **298**, 104-112 (2010).
42. Uechi, Y. & Uemura, R. Dominant influence of the humidity in the moisture source region on the ^{17}O-excess in precipitation on a subtropical island. *Earth Planet. Sci. Lett.* **513**, 20-28 (2019).
43. Sha, L. J. *et al.* Preparation of high-precision CO_2 with known triple oxygen isotope for oxygen isotope analysis. *Isot Environ Healt S* **57**, 443-456 (2021).
44. Hu, J., Emile‐Geay, J., Tabor, C., Nusbaumer, J. & Partin, J. Deciphering Oxygen Isotope Records From Chinese Speleothems With an Isotope‐Enabled Climate Model. *Paleoceanogr. Paleoclimatology* **34**, 2098-2112 (2019).
45. Hersbach, H., Bell, B., Berrisford, P., Biavati, G., Horányi, A., Muñoz Sabater, J., Nicolas, J., Peubey, C., Radu, R., Rozum, I., Schepers, D., Simmons, A., Soci, C., Dee, D. & Thépaut, J-N. ERA5 monthly averaged data on pressure levels from 1940 to present. Copernicus Climate Change Service (C3S) Climate Data Store (CDS), doi: 10.24381/cds.6860a573 (Accessed on 13-05-2023). (2023).
